# Calcined Co(II)-Triethylenetetramine, Co(II)- Polyaniline-Thiourea as the Cathode Catalyst of Proton Exchanged Membrane Fuel Cell

**DOI:** 10.3390/polym12123070

**Published:** 2020-12-21

**Authors:** Wen-Yao Huang, Li-Cheng Jheng, Tar-Hwa Hsieh, Ko-Shan Ho, Yen-Zen Wang, Yi-Jhun Gao, Po-Hao Tseng

**Affiliations:** 1 Department of Photonics, National Sun Yat-sen University, 70 Lienhai Rd., Kaohsiung 80424, Taiwan; wyhuang@faculty.nsysu.edu.tw; 2 Department of Chemical and Materials Engineering, National Kaohsiung University of Science and Technology, 415, Chien-Kuo Road, Kaohsiung 80782, Taiwan; lcjheng@nkust.edu.tw (L.-C.J.); thh@nkust.edu.tw (T.-H.H.); zxymzxym@gmail.com (Y.-J.G.); james090543@hotmail.com (P.-H.T.); 3 Department of Chemical and Materials Engineering, National Yun-Lin University of Science and Technology, Yun-Lin 64002, Taiwan

**Keywords:** co-catalyst, triethylenetetramine, thiourea, polyaniline, oxygen reduction reaction

## Abstract

Triethylenetetramine (TETA) and thiourea complexed Cobalt(II) (Co(II)) ions are used as cathode catalysts for proton exchanged membrane fuel cells (PEMFCs) under the protection of polyaniline (PANI) which can become a conducting medium after calcination. Fourier-transform infrared spectroscopy (FTIR) and X-ray photoelectron spectroscopy (XPS) spectra clearly reveal the presence of typical carbon nitride and sulfide bonds of the calcined Nitrogen (N)- or Sulfur (S)-doped co-catalysts. Clear (002) and (100) planes of carbon-related X-ray diffraction patterns are found for co-catalysts after calcination, related to the formation of a conducting medium after the calcination of PANI. An increasing intensity ratio of the D to G band of the Raman spectra reveal the doping of N and S elements. More porous surfaces of co-catalysts are found in scanning electronic microscopy (SEM) micropictures when prepared in the presence of both TETA and thiourea (CoNxSyC). Linear sweep voltammetry (LSV) curves show the highest reducing current to be 4 mAcm^−2^ at 1600 rpm for CoNxSyC, indicating the necessity for both N- and S-doping. The membrane electrode assemblies (MEA) prepared with the cathode made of CoNxSyC produces the highest maximum power density, close to 180 mW cm^−2^.

## 1. Introduction

Pt-catalysts for proton exchanged membrane fuel cells (PEMFCs) are carbon black (Vulcan XC-72) implanted with Pt nanoparticles (Pt/C) [1,2]. The carbon black (CB) subtracts function for both the conducting medium for the transportation of electrons from either cathode or anode, and O_2_ trappers with suitable sizes of pores. The CB comes from the calcination of common charcoal or carbon-containing materials. The so-called acetylene value, which represents the concentration of unsaturated, conjugated aromatics, is equivalent to conductivity.

When preparing a non-Pt catalyst such as iron- or cobalt-related compounds like the cathode catalyst of PEMFCs, a conducting medium needs to be constructed or added before it is doped by these metal ions. One of the convenient ways to obtain a large quantity of aromatics is to acquire a polymer whose monomer is also aromatic to combine with non-Pt catalyst precursors, before being calcined into catalysts. The monomer of polyaniline (PANI) is aniline, which has a benzene ring and amine and can be polymerized very easily in an acidic aqueous solution within several hours. Some studies indicate if Pt is reduced on the PANI matrix, the obtained Pt/PANi demonstrates better electrochemical properties [3,4,5,6,7]. The obtained PANI are mainly composed of aromatics and can be used as a nitrogen source as well, which can be used to dope the metal-catalysts after calcination.

Therefore, there is lots of research focusing on preparing cobalt-doped CB matrices by polymerizing PANI or complexes with cobalt salts with other Nitrogen (N)-containing chemicals and subjecting them to high temperature calcination [8,9] to become an N-doped co-catalyst (CoNxC) for the cathode catalyst of proton exchanged membrane fuel cells PEMFCs without the addition of conducting XC72. We prepare a covalently bonded network of CoNxCs using high temperature calcination to create a conducting medium through the carbonization of surrounding PANIs, simultaneously forming Co-Nx covalent bonds. The Co in the CoNxC (Co has an octahedral lattice with six coordinated sites) can coordinate with lesser or equivalent 4 N-s (Co-Nx-), while the other two empty sites can be used to attract O_2_, carrying out an oxygen reduction reaction (ORR) [10,11,12,13]. However, very few N-atoms can be provided by aniline monomers to form a complex with Cobalt(II) (Co(II)) before proceeding with the polymerization of PANI, which means the CoNxCs obtained directly from calcined PANI is not a good candidate to be cathode catalysts. Here, additional Triethylenetetramine (TETA), which can enclose and firmly catch Co(II) to become Co-N_4_ chelates [14,15,16], is used to increase the concentration of N-atoms and catch more O_2_, to improve the efficiency of CoNxC as a cathode catalyst of PEMFC. However, the formed N-doped compounds composed of pyridinic, graphitic, and pyrrolic N-atoms all have very flat structures so, the surfaces of N-doped CoNxC catalysts will be very smooth due to the similar size of the doping N-atoms with the C-atoms of the matrix and, thus, cannot provide enough traps to catch O_2_ molecules. Replacing some of the N-atoms by S-atoms will increase the roughness of the formed CoSyC (S-doped co-catalyst) surfaces with the significantly bigger size of the S-atoms compared to the C-atoms [17]. Besides, there is a synergistic effect between Co-S and the carbon matrix [17,18,19,20,21]. The S-atoms will be added in the form of thiourea, which does not include only S-atoms but carries two amines to provide more N-atoms for the co-catalysts.

During our study, the CoNxCs will be prepared by complexing Co ions with TETA before the polymerization of the covering PANI. Regarding the CoSyC system, the TETA will be replaced with thiourea. Eventually, both TETA and thiourea will be used during the preparation of the co-catalysts (CoNxSyC). Moreover, the coordinated atoms with the Co-lattice will include both N- and S-atoms to enhance the oxygen reduction reaction (ORR) in the cathode of the proton exchanged membrane fuel cells (PEMFCs). These three types of co-catalysts will be characterized, and their electrochemical properties will be measured and compared to find out the one most suited for the cathode catalyst for PEMFCs.

## 2. Experimental

### 2.1. Materials

Aniline (Tokyo Kasei Kogyo Co., Ltd., Tokyo, Japan), hydrochloric acid (Scharlau, Spain), ammonium hydroxide (Fisher Sci., Bridgewater, NJ, USA), ammonium persulfate (J.T.Baker, Phillipsburg, NJ, USA), Cobalt(II)nitrate hexahydrate (Co(NO_3_)_2_·6H_2_O, Showa, Okayama, Japan), triethylenetetramine (C_6_H_18_N_4,_ Acros Organics, NJ, USA), thiourea (CS(NH_2_)_2_, Acros Organics, Morris Plains, NJ, USA), Vulcan XC-72 (Cabot, MA, USA).

### 2.2. Preparation of CoNxC Catalyst

One gram of Co(NO_3_)_2_·6H_2_O and 1.5 mL Triethylenetetramine (TETA) was mixed with 50 mL de-ionized water and 5 g of aniline monomers, with stirring to become uniform. Ten milliliters of 1 M HCl(aq) solution was introduced into the mixture and stirred again, followed by the addition of a solution of 10 g ammonium persulfate (APS) in 15 g de-ionized water to start the polymerization until the mixed solution became dark green (about 1.5 h). After the polymerization, the solvents of the mixture were removed in a vacuum oven at 80 °C and the precursor of the CoNxC catalyst was obtained. The precursor was heated to 800 °C at 10 °C min^−1^ increments, maintained for 1 h. in the argon atmosphere, then cooled to room temperature (RT). The impurities and magnetic materials of the obtained CoNxCs were removed by washing in 1 M H_2_SO_4_(aq) at 80 °C for 3 h, followed by drying in a vacuum oven at 60 °C. The crude CoNxC was further calcined to 700 °C in N_2_ and NH_3_ atmospheres again at 10 °C min^−1^ increments, washed again in 1 M H_2_SO_4_(aq) at 80 °C for 3 h, followed by drying in a vacuum oven at 60 °C.

CoSyC was prepared with the same procedures except 2 g of thiourea were used to substitute 1 g of Triethylenetetramine (TETA). CoNxSyC was obtained using both 1 g TETA and 2 g thiourea. The comparison experiment of preparing the Co-C catalyst was conducted with the same steps except 1 g of Vulcan XC-72 was used to replace TETA and thiourea and it was not covered by polymerizing polyaniline PANI.

### 2.3. Fourier-Transform Infrared Spectroscopy (FTIR)

The main functional groups of various co-catalysts were assigned from Fourier-transform infrared spectroscopy (FTIR) spectra which were recorded on an IFS3000 v/s FTIR spectrometer (Bruker, Ettlingen, Germany) at room temperature with a resolution of 4 cm^−1^ and 16 scanning steps.

### 2.4. X-ray Photoelectron Spectroscopy (XPS)

The different binding energy spectra of N1s and S2p of various co-catalysts were used to characterize the percentages of pyridinic, pyrrolic, graphitic Ns, C–S–C, -C=S, Co–S–C, and C–S–N…etc., after calcination by an X-ray photoelectron spectroscopy (XPS) instrument from Fison (VG)-Escalab 210 using an Al Ka X-ray source at 1486.6 eV. The pressure in the chamber was maintained under 10–6 Pa or lower during the measurement. A tablet-like sample was prepared by a stapler.

### 2.5. WXRD (Wide Angle X-ray Diffraction: Powder X-ray Diffraction)

A copper target (Cu-Kα) Rigaku X-ray source with a wavelength of 1.5402 Å was used for X-ray diffraction. The scanning angle (2θ) travelled from 10° to 90° with a voltage of 40 kV and a current of 30 mA, operated at 1° min^−1^ increments.

### 2.6. Raman Spectroscopy

The Raman spectra of all samples were obtained from a Raman spectrometer (TRIAX 320, HOBRIA, Kyoto, Japan).

### 2.7. Scanning Electronic Microscopy (SEM)

The sizes and morphologies of all co-catalysts were characterized by scanning electronic microscopy (SEM) (field emission gun scanning electron microscope, AURIGAFE, Zeiss).

### 2.8. Electrochemical Characterization

#### 2.8.1. Linear Sweep Voltammetry (LSV)

The performance of the electrocatalyst support was tested with a three-electrode system. The round working electrode with an area of 1.5 cm^2^ was prepared as follows. Ag/AgCl and carbon graphite were used as the reference and relative electrodes, respectively. The electrochemical test was carried out in a potentiostat/galvanostat (Autolab-PGSTAT 30 Eco Chemie, Utrecht, The Netherlands) in 0.5 M of an H_2_SO_4_ solution, and linear sweep voltammetry (LSV) curves were obtained with scanning potential from −0.2 to 1.0 V at a sweeping rate of 50 mV s^−1^. The catalyst ink was prepared by mixing 3 mg of support powder in isopropanol and stirring until it became uniform. Subsequently, 5% Nafion™ solution was added into the mixture as a binder and the mixture was ultra-sonicated for 1 h. The obtained ink was uniformly spray-coated on the carbon paper for the LSV test.

The current-potential polarization curves obtained from the LSV of the various co-catalysts were measured using a rotating-disk electrode (RDE) operated at 900, 1200, 1600, 2500, and 3600 rpm in O_2_-saturated 0.5 M H_2_SO_4_, respectively. The reduced current densities were recorded within the measured voltage range (0.0–1.3 V) at 1600 rpm.

#### 2.8.2. Membrane Electrode Assemblies (MEA) Preparation

A Nafion™ 212 sheet purchased from Ion Power Inc., (New Castle, DE, USA) was used as the proton exchange membranes. To remove the surface organic impurities and to convert the membranes into protonated form, the Nafion™-212 (4 × 4 cm^2^) membrane was treated at 70 °C in a 5 wt.% H_2_O_2_ aqueous solution for 1 h, followed by submerging in a 1 M H_2_SO_4_ solution for 1 h. Subsequently the treated membranes were dipped in distilled water for 15 min and stored in de-ionized water. The catalyst inks were prepared by mixing 20 mg of co-catalyst powders in isopropanol with mechanical stirring until they became uniform before a 5% Nafion™ solution was added. Eventually, the catalyst mixture was ultra-sonicated for 1 h, followed by coating on both sides of the treated Nafion™ sheet dropwise as anode and cathode electrodes (2 × 2 cm^2^), respectively, and hot-pressed at 140 °C with a pressure force of 70 kg cm^−2^ for 5 min to obtain the membrane electrode assemblies (MEAs).

### 2.9. Single-Cell Performance Testing

The membrane electrode assemblies (MEAs) were installed in a fuel cell test station for testing using the single-cell test equipment (model FCED-P50; Asia Pacific Fuel Cell Technologies, Ltd., Miaoli, Taiwan). The active cell area was 2 × 2 cm^2^. The temperatures of the anode, cell, cathode, and humidifying gas were all maintained at around 70 °C. The flow rates of the anode input H_2_ and the cathode input O_2_ fuels were set at 200 and 100 mL·min^−1^, respectively, based on stoichiometry. To test the electrochemical performance of the co-catalyst in the individual MEAs, both the current-voltage (C-V) curves and output powers were measured.

## 3. Results and Discussion

### 3.1. Fourier-Transform Infrared Spectroscopy (FTIR) Spectra

Most of the Cobalt(II) (Co(II)) are captured inside the porphyrin, which is made of heterocyclic pyrroles through very tedious procedures [15] before being subjected to calcination. During this study, Co(II) was simply combined with the amino groups of Triethylenetetramine (TETA) or thiourea, saving the trouble of preparing the complicated porphyrin-Co salts. Moreover, more Nitrogen (N) elements can be provided by both TETA and thiourea, the Sulphur (S) element is provided by thiourea readily, and the S-doped compounds are usually prepared with vaporized sulfur through a chemical vapor deposition (CVD) process [18,19].

**Scheme 1:**
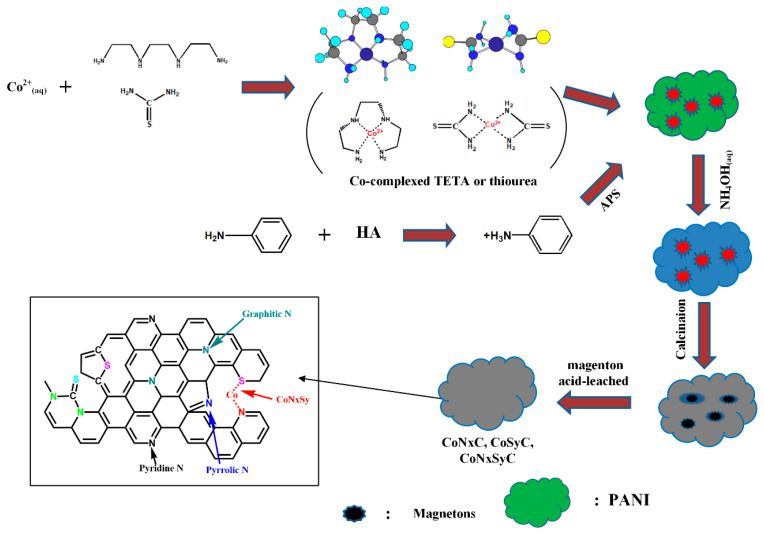
Preparing diagram of various co-catalysts.

Unlike thiourea, which needs two members to capture one Co^+2^, one TETA molecule can form stable complexes with Co^+2^ by enclosing, (Scheme 1).

**Scheme 2:**
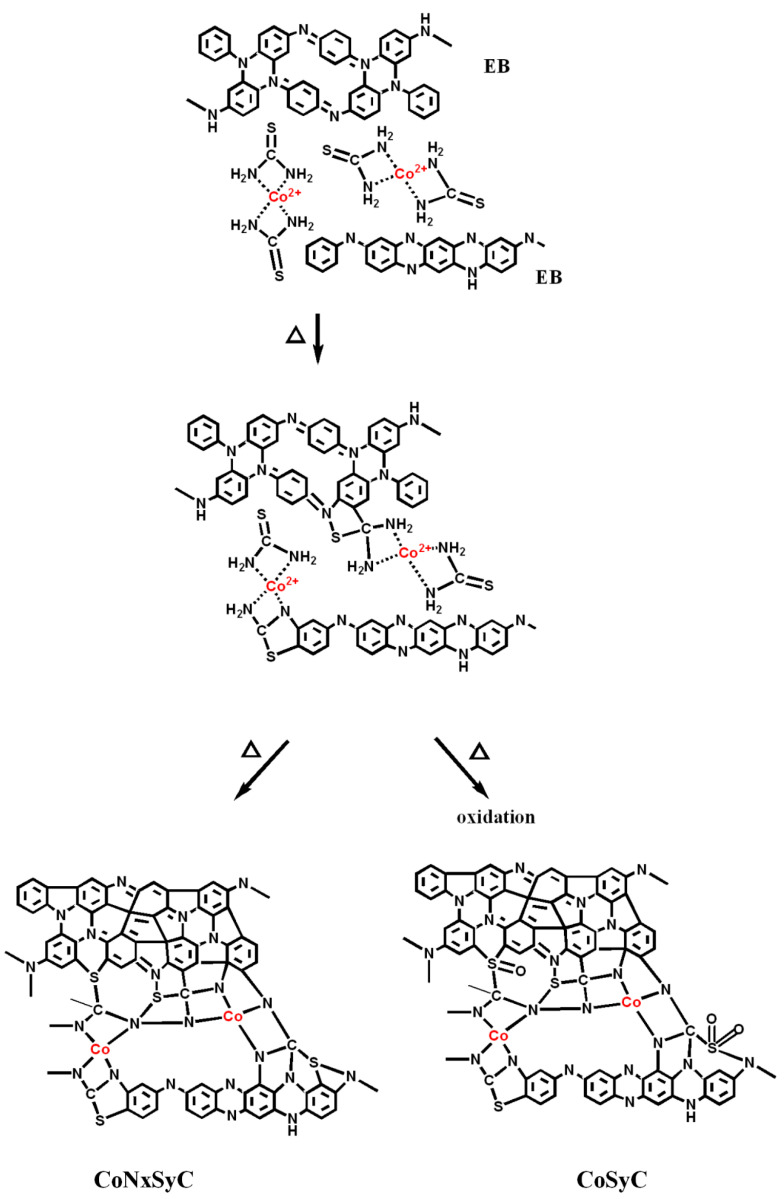
Electrocatalytic mechanism of N-, S-doped co-catalysts.

**Figure 1:**
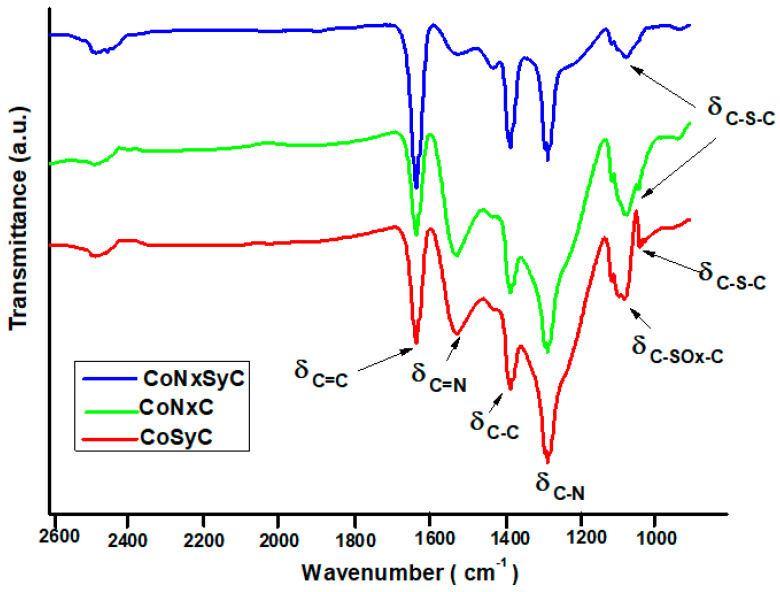
Fourier-transform infrared spectroscopy (FTIR) spectra of various co-catalysts.

When only thiourea was combining with Co^2+^ ions before being covered by polyaniline (PANI) via polymerization and subjected to two-stage calcination, significant characteristic peaks of C=N, C=C, C–N, and C–C could be seen, Figure 1 (CoSyC). Besides, thio-carbon chain (C–S–C) also are found around 900 cm^−1^, resulting from the C=S of thiourea. It indicates sulfur is able to incorporate into the Co–C network matrix and become CoSyC. An additional peak is clearly seen above 1000 cm^−1^, however, contributed by the oxidation of sulfur, leading to the formation of sulfoxide (–SOx–), described in Scheme 2. The lone-pair electron of sulfur of –SOx– was already occupied by oxygen and lost the ability to capture O_2_ in the cathode during the performance of the fuel cell. Likewise, if we replaced thiourea with TETA, the obtained CoNxC still demonstrated the feature peaks of an N-doped structure in its IR-spectrum (Figure 1). The formation of the C–S–C group originated from the sulfur of the APS, which was used as an initiator for the polymerization of the polyaniline (PANI).

Interestingly, when both thiourea and TETA were used as Co^2+^ ion capturers, the formed CoNXSyC did not demonstrate any sulfoxide peak, revealing that the introduction of TETA can capture more Co^2+^ but also form an N-doped matrix to protect the sulfur from oxidation during high temperature calcination. It seems both thiourea and TETA play their roles in improving the oxygen reduction reaction (ORR) of Co–C catalysts in the cathode.

### 3.2. X-ray Photoelectron Spectroscopy (XPS)

**Scheme 3:**
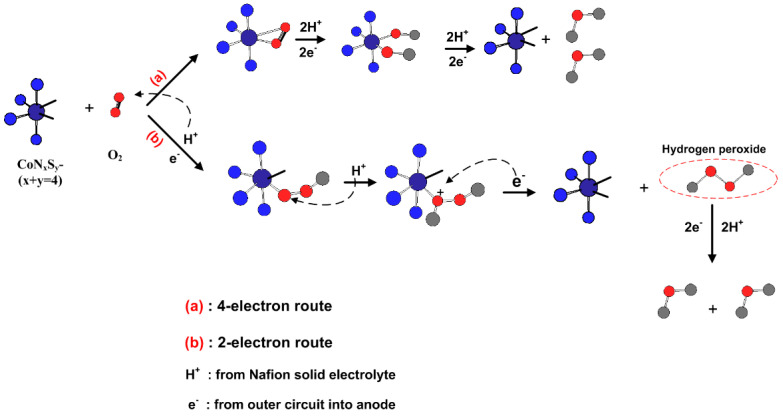
ORR in the cathode catalyzed by CoNxSyC.

The active sites of co-catalysts are formed of octahedral crystals with six coordinate sites, according to Scheme 2. Usually, in this study, less than four of them were occupied by polar dopants like nitrogen or sulfur, leaving the rest of the sites to the incoming O_2_ and carrying out the oxygen reduction reaction (ORR), as described in Scheme 2. However, two possible routes were adopted by ORR. One was the 4-electron route which allowed the direct reduction of O_2_ into water (Scheme 3a). The other took a two-step route where, before the formation of water, during the intermediate step, hydrogen peroxide was produced, as seen in Scheme 3b (2-electron route). The ORR was belayed, in other words, and less than four electrons were transferred during ORR, resulting in the incompleteness of ORR and the production of unexpected H_2_O_2_.

**Figure 2:**
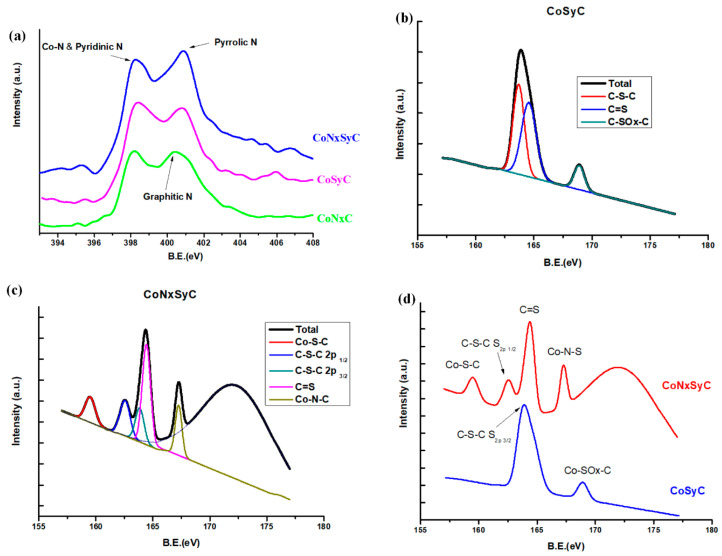
X-ray photoelectron spectroscopy (XPS) spectra of (**a**) N1s of all co-catalysts (**b**) S2p of CoSyC (**c**) S_2p_ of CoNxSyC (**d**) Comparing S_2p_ of CoSyC with CoNxSyC.

When Co is doped by polar nitrogen, its catalyzing ability can be enhanced and most of the oxygen reduction reactions (ORRs) will be a 4-electron reaction. Here, polyaniline (PANI) was not used only as the C-source to form a conducting matrix after calcination, but also as the Nitrogen (N)-source allowing the formation of an N-doped co-catalyst (CoNxC) after calcination. To increase the N-composition in the CoNxC, Triethylenetetramine (TETA) was first complexed with Co ions before the polymerization of polyaniline (PANI). However, there are several forms of N-doping after calcination. They could be pyrrolic, graphitic, pyridinic [22,23,24], and Co-doped nitrogen, and each type of nitride can contribute to the enhancing catalysis of Co-compounds. The presence of N in the carbon matrix of a co-catalyst can increase the polarity of the non-polar conducting carbon matrix and improve the O_2_ adsorption efficiency, leading to the fast oxygen reduction reaction (ORR). These different forms of nitride can be characterized by the N_1s_ X-ray photoelectron spectroscopy (XPS) spectra demonstrated in Figure 2a which illustrates the N_1s_ absorption peaks obtained from various Co-compounds.

Additionally, the incorporation of sulfur in the co-catalysts can improve the ORR as well. Mostly, the sulfides presented in the carbon matrix will be in C–S–C form if calcination is conducted in a non-oxygenized environment, in which two lone pairs of sulfide are retained. The additional two lone pair electrons of sulfide also can increase the adsorption of O_2_ in the cathode. Furthermore, the CoNxC matrix still provides a very planar structure of the co-catalysts since the size of N is similar to that of C and fewer O_2_ gas molecules can be trapped on the smooth surfaces of the catalysts. The introduction of the larger S can induce a rougher surface on the CoNxC, which catches more O_2_ in the cathode.

The different forms of sulfide in S-doped (CoSyC) and N-S-codoped co-catalysts (CoNxSyC) are examined by the S2p spectra of XPS in Figure 2b,d. When the co-catalyst was only doped with S via the addition of thiourea during preparation, the S2p demonstrated only C=S, C–S–C and C–SOx–C. Although the S indeed was doped into the CoNxC matrix, part of the C–S–C groups were oxidized and became sulfoxides, on which the lone pairs of S were occupied by the oxygen, losing the power to attract O_2_ gas.

When both thiourea and TETA were present in the preparation of the co-catalysts, the obtained CoNxSyC demonstrated no oxidation of the C–S–C group, the peak of C-SOx-C disappeared, and additional peaks of Co–N–S and Co–S–C were found, indicating S was deeply doped into the CoNxC structure and covalently bonded to N and Co. It seems that the formation of the CoNxC matrix can protect the C–S–C groups of the CoSyC matrix from oxidation. Briefly, both thiourea and TETA are necessary to prepare a co-catalyst to improve ORR. The evaluation of ORR will be examined later by comparing the reducing currents of CoNxC, CoSyC, and CoNxSyC.

### 3.3. Wide Angle X-ray Diffraction: Powder X-ray Diffraction (WXRD)

**Figure 3:**
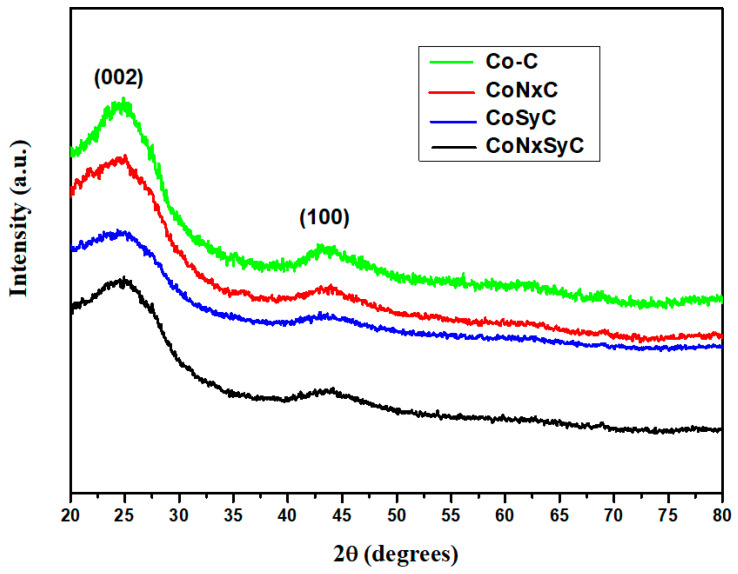
X-ray diffraction patterns of various co-catalysts.

Co ions were directly mixed with Vulcan-XC72 carbon black without doping with any compound before two-stage calcination (Co-C) took place. The X-ray diffraction (XRD) patterns of all the co-catalysts demonstrated similar diffraction peaks to Co-C, in Figure 3, indicating most of the carbons originated from the polyaniline (PANI) matrix (benzene rings) which were carbonized and formed an ordered, graphite-like conducting matrix similar to Co-C. It is not necessary, in other words, to put an additional conducting medium like Vulcan-XC-72 into the preparation of the catalyst via calcination. The (002) plane peak represents the amorphous carbonaceous interlayer [25,26] and (100) was contributed by crystalline carbon [27,28]. Therefore, the intensity ratio of I_100_/I_002_ can be used to illustrate the crystallinity of the co-catalysts. Table 1 lists the ratios of various co-catalysts. The Co-C has the highest I_100_/I_002_ ratio of 0.459, and the ratios for CoNxC and CoSyC are 0.432 and 0.427, respectively. This reveals that a more ordered carbon structure can be constructed by calcination if no dopant like Nitrogen (N) or Sulphur (S) is present. Doping by N can obtain a more ordered structure than S-doping since it has a similar size to C atoms, and the larger S would destroy the planarity of the obtained network structure after calcination. When both N and S were doped into the Co–C matrix, the crystalline was destroyed significantly and the I_100_/I_002_ ratio decreased to 0.412 (Table 1) due to the mismatching sizes of S and N in the carbon matrix. No significant crystalline peak related to the Co element or Co oxide is seen in Figure 3, revealing most of them were already removed by acid leaching adopted before the second stage of calcination was conducted in the mixed gases of N_2_ and NH_3_.

### 3.4. Raman Spectroscopy

**Figure 4:**
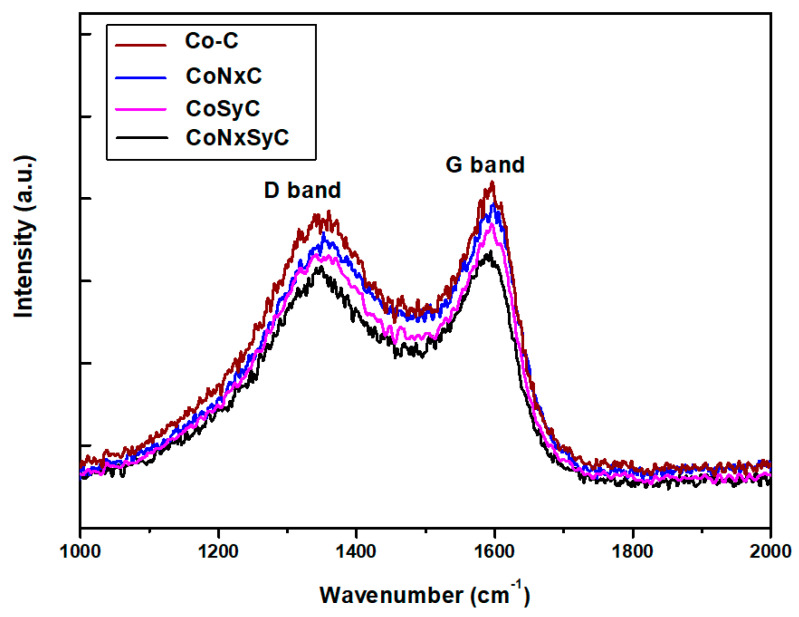
Raman spectra of various co-catalysts.

Another approach to identify the crystallinity (conductivity) of the calcined carbon matrix of co-catalysts is to check the ratio of the D and G bands of the Raman spectroscopy, shown in Figure 4. The D band (~1350 cm^−1^) can be related to the sp3 orbitals of the C–C bonds, and contributes to the amorphous part of the carbon matrix due to its mobile single bond. The G band (~1590 cm^−1^) comes from the unsaturated C=C bonds which can be rotated, and results in the crystalline part of the carbon matrix in the form of aromatics. Therefore, the intensity ratio of these two types of bands, I_D_/I_G_ is inversely similar to that of the I_100_/I_002_ ratio. According to Table 1 and Figure 4, Co–C demonstrates the lowest I_D_/I_G_ value of 0.970 due to the higher crystallinity and concentration of the C=C bonds. The crystallinity was slightly increased to 0.973 after nitrogen was doped into the Co–C matrix (CoNxC). It continually increased to 0.978 when doped nitrogen was replaced with doped sulfur (CoSyC). Eventually, we obtained the highest value of 0.987 when both nitrogen and sulfur were doped into the Co–C matrix (CoNxSyC). Although the crystallinity of Co–C was partly destroyed by the doping of nitrogen or sulfur, the I_D_/I_G_ values of all co-catalysts were all below one, revealing the concentration of the crystalline region was still over the amorphous one to provide a stable conducting medium for the performance of the oxygen reduction reaction (ORR) in the cathode.

### 3.5. Scanning Electronic Microscopy (SEM)

**Figure 5:**
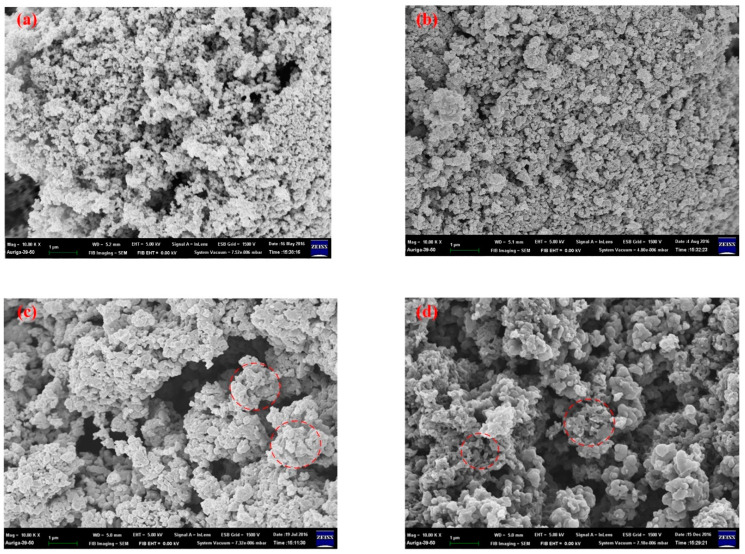
Scanning electronic microscopy (SEM) micropictures of (**a**) Co–C (**b**) CoNxC (**c**) CoSyC (**d**) CoNxSyC.

The micropicture of scanning electronic microscopy (SEM) in Figure 5a demonstrates lots of accumulated, individual nanoparticles of carbon black after calcination, and no fusion phenomenon is seen for the particles. The distribution pattern of these nanoparticles is good for the oxygen reduction reaction (ORR). However, not significant ORR is found, which will be discussed in the electrochemical sections. The almost non-polar nature of the Co–C cannot attract the O_2_ fuel in the cathode, illustrating the importance of introducing polar nitrogen or sulfur into the matrix to improve the polarity, even though the perfect morphologies could be changed after doping.

The morphologies become denser after doping with nitrogen (CoNxC), as seen in Figure 5b and some of the nanoparticles were stuck together. No significant coral-like structure, which originates from calcined polyaniline (PANI), could be found. The porosity of the CoNxC is smaller compared to Co–C. When nitrogen is substituted with sulfur, the morphologies changed a lot, as seen in Figure 5c. Not just huge cave-like holes are observable, but the particles became bigger and some tiny sections of entangled PANI nanofibers are found. When both nitrogen and sulfur were doped into Co–C (CoNxSyC), the morphologies developed into more porous structures with lots of medium-sized holes created and more entangled short nanofibers of PANI randomly distributed in the entire area. The presence of PANI nanofibers of CoNxSyC after the two-stage high temperature calcination, plus strong acid leaching, indicates the there are lots of N- or S-doped regions remaining in the co-catalysts under the protection of PANI nanofibers.

### 3.6. Oxygen Reduction Reaction (ORR) Performance

#### Linear Sweep Voltammetry (LSV) Curves

**Figure 6:**
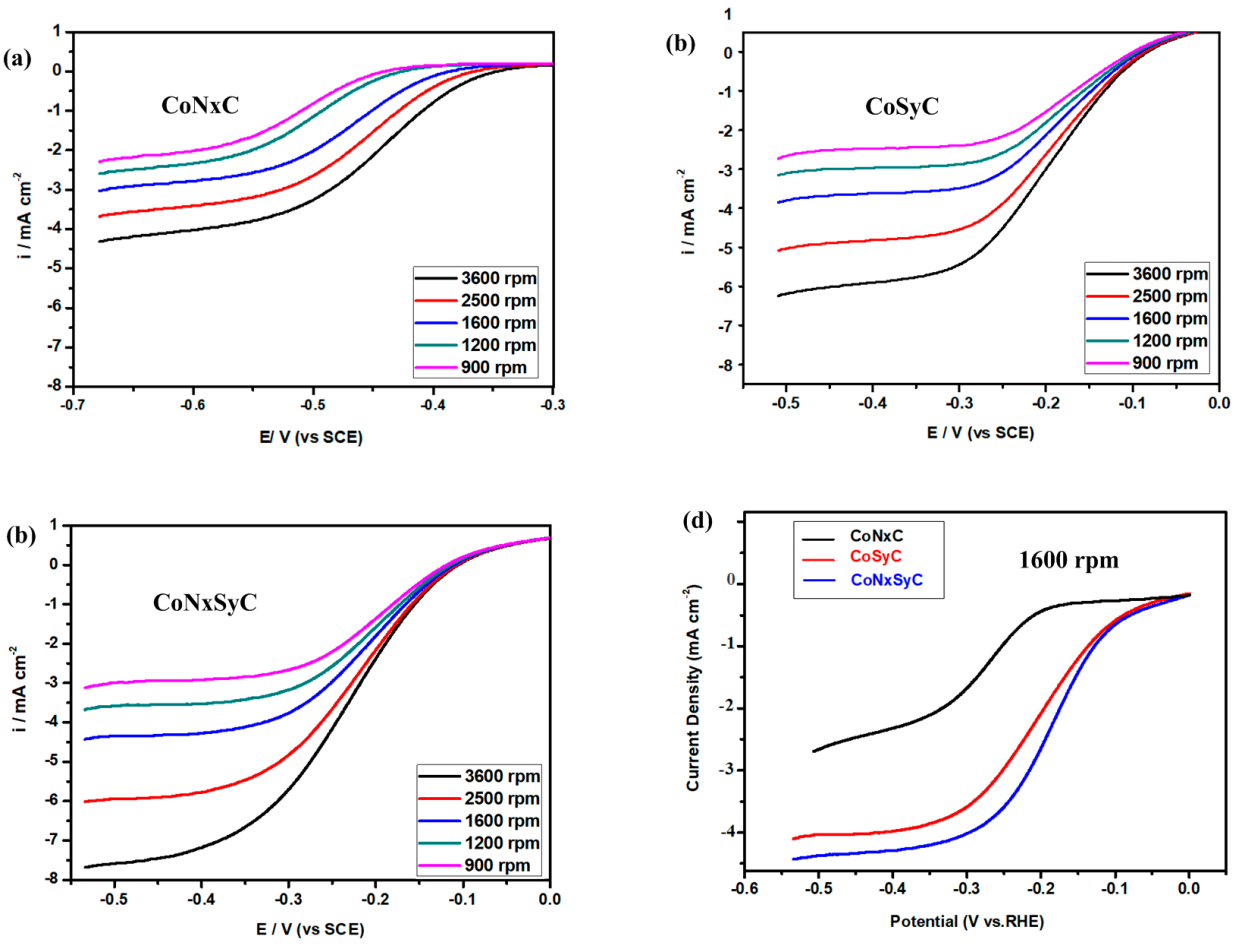
Linear sweep voltammetry (LSV) curves at all rpms of (**a**) CoNxC (**b**) CoSyC (**c**) CoNxSyC (**d**) LSV curves at 1600 rpm of all catalysts.

The reduced current density was controlled by the concentration of the input of O_2_ in the cathode in the beginning of the redox reaction, the so-called kinetic current (I_kin_), which remained almost the same when rotating speeds were increased during the initial stage, according to Figure 6a,c. When the potential was decreased, the current density significantly decreased to a plateau value called the diffusion current (I_dif_); in other words, the reducing current now was dependent on the diffusion rate of O_2_, not the concentration anymore. The I_dif_ values increased with rotating speeds.

The linear sweep voltammetry (LSV) curves illustrated in Figure 6a,c were obtained from the RDE conducting at different speeds. The presence of coordinated Nitrogen (N) or Sulphur (S) was similar to the Ziegler Natta catalysts used in the coordination polymerization applied to products PP and HDPE, which can absorb unsaturated monomers (CH_2_=CH_2_) equivalent to O=O, to the empty sites of CoNxSyC catalysts (Scheme 2) for the oxygen reduction reaction (ORR). Figure 6d compares the LSV curves of all co-catalysts at 1600 rpm, and the CoNxSy which has a reduced current comparable to the N- and/or S-doped graphene [16] prepared with complicated procedures. Therefore, the presence of both N- and S-atoms did improve the capture of O_2_ by the co-catalysts, and more ORRs were carried out in the 4-electron route during which less H_2_O_2_ was formed and a higher reducing current could be obtained.

### 3.7. Single Cell Testing

**Figure 7:**
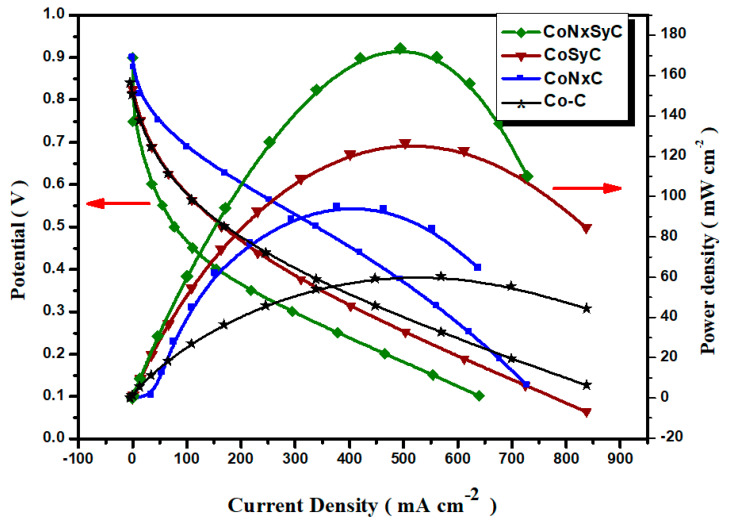
Power and current densities of single cells made of various co-catalyst cathodes.

The single cell made of the Co–C cathode catalyst prepared by mixing CB (XC-72) with Co ions before calcination demonstrates a lower maximum power (less than 60 mW cm^−2^) or current density, according to Figure 7. When only Co ions were mixed with CB (XC-72) in the absence of polyaniline (PANI) and not doped by Nitrogen (N)- or Sulphur (S)-atoms, the obtained single cell produced a maximum power density below 60 mW cm^−2^, according to Figure 7, even though the structure of the calcined XC-72 was more porous, and both its crystallinity and conductivity were higher than the rest of the co-catalysts. When PANI and Triethylenetetramine (TETA) were joined in the preparation of the co-catalyst (CoNxC), the single cell demonstrated a higher maximum power density of 93 mW cm^−2^. When TETA was replaced by thiourea, the obtained single cell produced a higher maximum power density of 125 mW cm^−2^. Actually, it was still an N-doped co-catalyst since the Co-thiourea complexes were covered with PANI before calcination. Therefore, it needed both N- and S-atoms in the networks of the co-catalyst for a higher power density. When both TETA and thiourea were used to combine with Co ions before polymerization of the PANI and its subsequent calcination, the resultant single cell demonstrated the highest maximum power density of 171 mW cm^−2^, indicating the N-atom concentration was too low for CoSyC and more N-atoms could be significantly increased via the complexation between the Co ions and TETA before calcination.

## 4. Conclusions

The covering of the co-catalyst by polyaniline (PANI) can provide both Nitrogen (N)-atoms and a conducting carbon matrix through calcination in an inert atmosphere, demonstrated by X-ray and Raman spectra.

The smooth surfaces of CoNxC made of pyridinic, graphitic, and pyrrolic nitrogen can be made rougher by doping with Sulphur (S)-atoms from thiourea, which also can improve the capture of O_2_ in the cathode by the additional lone pair electrons. The presence of both N- and S-atoms as the cathode catalysts of proton exchanged membrane fuel cells (PEMFC) can significantly improve the oxygen reduction reaction (ORR) in the cathode by demonstrating clear reduction peaks in the C-V curves and producing a higher reducing current density in the linear sweep voltammetry (LSV) curves. The single cell prepared with a cathode made of both N- and S-doped co-catalysts (CoNxSyC) produces a high maximum power density of 171 mW cm^−2^, which is a pretty high value for prepared co-catalysts.

Henceforth, both Triethylenetetramine (TETA) and different types of S-containing compounds will be used to complex with Co ions before PANI polymerization and subsequent two-stage calcination to obtain new types of CoNxSyC cathode catalysts for PEMFC, to achieve more active ORR, and better electrochemical performance.

## Figures and Tables

**Table 1:** Relative intensity ratios of plane (100)/(002) and D/G band.

	I_100_/I_002_^(a)^	I_D_/I_G_^(b)^
**Co–C**	0.459	0.970
**CoNxC**	0.432	0.973
**CoSyC**	0.427	0.978
**CoNxSyC**	0.412	0.987

(a): obtained from the X-ray diffraction patterns. (b): obtained from the Raman spectroscopy.
